# A novel 1p13.2 deletion associates with neurodevelopmental disorders in a three-generation pedigree

**DOI:** 10.1186/s12920-023-01534-7

**Published:** 2023-05-23

**Authors:** Lihua Yu, Hongke Ding, Min Liu, Ling Liu, Qi Zhang, Jian Lu, Fangfang Guo, Yan Zhang

**Affiliations:** 1 Medical Genetics Centre, Guangdong Women and Children Hospital, Guangzhou, Guangdong China; 2 Prenatal diagnostic center, Huizhou No2 Maternal and Children’s Healthcare Hospital, Huizhou, China

**Keywords:** 1p13.2 deletion, Neurodevelopmental disorders, Whole-exome sequencing, Molecular diagnosis

## Abstract

**Background:**

A multitude of studies have highlighted that copy number variants (CNVs) are associated with neurodevelopmental disorders (NDDs) characterized by a wide range of clinical characteristics. Benefiting from CNV calling from WES data, WES has emerged as a more powerful and cost-effective molecular diagnostic tool, which has been widely used for the diagnosis of genetic diseases, especially NDDs. To our knowledge, isolated deletions on chromosome 1p13.2 are rare. To date, only a few patients were reported with 1p13.2 deletions and most of them were sporadic. Besides, the correlation between 1p13.2 deletions and NDDs remained unclear.

**Case presentation:**

Here, we first reported five members in a three-generation Chinese family who presented with NDDs and carried a novel 1.41 Mb heterozygous 1p13.2 deletion with precise breakpoints. The diagnostic deletion contained 12 protein-coding genes and was observed to segregate with NDDs among the members of our reported family. Whether those genes contribute to the patient’s phenotypes is still inconclusive.

**Conclusions:**

We hypothesized that the NDD phenotype of our patients was caused by the diagnostic 1p13.2 deletion. However, further in-depth functional experiments are still needed to establish a 1p13.2 deletion-NDDs relationship. Our study might supplement the spectrum of 1p13.2 deletion-NDDs.

**Supplementary Information:**

The online version contains supplementary material available at 10.1186/s12920-023-01534-7.

## Background

Neurodevelopmental disorders (NDDs), encompassing a wide range of clinical phenotypes, include but are not limited to intellectual disability (ID), autism spectrum disorder (ASD), schizophrenia (SCZ), attention deficit hyperactivity disorder (ADHD), developmental delay (DD), epilepsy, and specific learning disorders (SLD) [1–3]. A multitude of studies have highlighted that copy number variants (CNVs) are associated with NDDs [1, 4–6]. Nevertheless, in most of the observed clinical cases, the pathogenicity of CNVs, especially rare CNVs, remains unclear.

Nowadays, in addition to the conventional detection of single nucleotide variants (SNVs) and small insertions/deletions (Indels), exome-based CNV analysis is another important application of whole-exome sequencing (WES) [7–10]. Here, by trio WES, a novel 1.41 Mb heterozygous 1p13.2 deletion was detected in a patient with NDDs, and the potentially pathogenic SNVs/Indels related to the patients’ phenotypes were excluded.

To the best of our understanding, isolated deletions on chromosome 1p13.2 are uncommon; thus far, only five published pieces of literature have reported that patients with 1p13.2 deletions displayed NDDs [11–15]. But few patients have a family history, and the definite evidence of the association between 1p13.2 deletion and NDDs still needs further functional studies.

## Case presentation

**Fig. 1:**
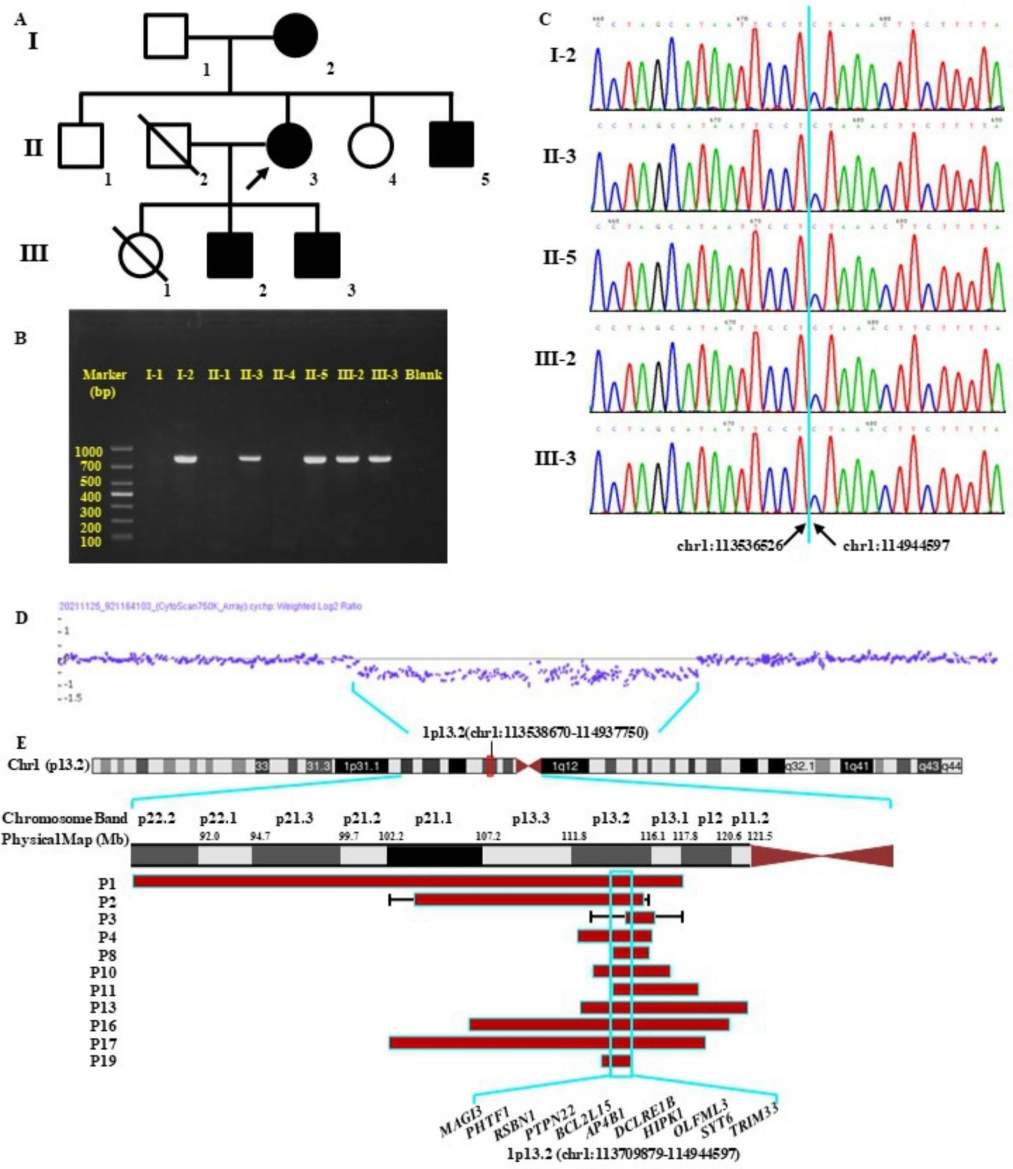
**(A)** Pedigree for this three-generation family. Arrows indicate the proband. Solid circles/squares indicate individuals with a heterozygous 1p13.2 deletion. **(B)** Electrophoresis of gap-PCR products. The presence of an 836 bp band indicated that the corresponding individuals in the proband’s family carried the 1p13.2 deletion. **(C)** The accurate deletion breakpoints for the patients (I-2, II-3, II-5, III-2 and III-3), were validated by Sanger sequencing. A 1,408,072 bp deletion at 1p13.2 was identified (chr1:113,536,526–114,944,597). **(D)** The 1.31 Mb deletion at 1p13.2 (chr1: 113,538,670–114,937,750) of our proband was detected by chromosomal microarray analysis. **(E)** Schematic representation of the deletion region in our patients and those included for further analysis, with overlapping deletion regions at 1p13.2. Ideogram of chromosome 1, physical map and deleted regions are referred to their placement on the UCSC Genome Browser on Human (GRCh37/hg19).

The Guangdong Women and Children Hospital Medical Ethics Committee approved the study, and informed consent was obtained. A 34-year-old gravida 3, para 3 (G3P3) Chinese woman, with neurodevelopmental disability, underwent genetic counseling in Huizhou No.2 Maternal and Children’s Healthcare Hospital and was referred to Medical Genetic Centre in Guangdong Women and Children Hospital for further genetic testing. The proband had a mild facial appearance with full eyebrows, hypertelorism, ptosis, low-set ears, upturned nose, overbite, slightly open mouth and webbed neck, pictures of whom was not authorized by her family. She could sit, walk and run without support, but her gross motor milestones were unclear. No other physical abnormalities were observed during genetic counseling. She was noted to present ID, studied in a special education school in childhood, and could not perform instrumental activities of daily living (including cooking, cleaning, washing clothes, making telephone calls, financial management, and so on). Her receptive language and speech were significantly delayed, and she communicated with others just by a few words. Activities of daily living, such as dressing, eating, bathing and toileting, could be performed by herself. The patient had not any epilepsy history and denied behavioral problems, including ADHD, motor stereotypies, self-injury behavior, aggressive behavior and social anxiety. Four members of the proband’s family, including her mother, her younger brother and two sons, had similar symptoms. In contrast, her elder brother, sister and daughter (who died in a traffic accident) had normal phenotypes (Fig. 1A). After genetic counseling, the proband and her family finally decided to receive trio WES, and also they accepted the screening for fragile X syndrome for her elder son. Further mental and behavioral assessments and brain Magnetic Resonance Imaging (MRI) examinations could not be conducted in the family.

## Methods

### Trio whole-exome sequencing

Peripheral blood samples (2 mL) were drawn from the proband and her family. Genomic DNA samples were isolated using RelaxGene Blood DNA System (Tiangen, Beijing, China), following the manufacturer’s instruction.

Trio WES was performed on the proband and her parents. The target genomic regions were captured by hybridizing the genomic DNA sample library with the xGen® Exome Research Panel v1.0 (IDT, USA). High-throughput sequencing was then performed on the Illumina NovaSeq6000 platform (Illumina, San Diego, CA, USA) with 150 bp paired-end reads. Briefly, raw sequencing reads were aligned to the human reference genome hg19/GRCh37 via BWA [16]. Variants and indels were called by the HaplotypeCaller tool of GATK [17]. VEP was employed to identify the effect of all variants followed by variant annotation with AnnoVar [18]. The annotated variants were filtrated in an in-house stepwise protocol as shown in the Supplemental method. Notably, each variant was compared against public databases, including gnomAD, 1000 genomes project, NHLBI Exome Sequencing Project 6500 (ESP6500), and Exome Aggregation Consortium (ExAC), to achieve allele frequency in the general population. In terms of possible influence on the protein function, candidate variants were evaluated by VarCards [19]. The classification of candidate variants was interpreted based on the American College of Medical Genetics (ACMG) guidelines 2015 [20].

Copy number variants (CNVs) were called by CNVkit [21]. To create a stable and reliable CNV reference, in-house 80 samples without any CNV events larger than 1Mbp over the same sequencing protocol were selected for reference training in an iterative manner. AnnotSV and its annotation databases were locally installed to annotate detected CNV events for each tested sample for following clinical interpretation [22]. The Database of Genomic Variants (DGV) (http://dgv.tcag.ca/dgv/app/home), the DatabasE of genomiC varIation and Phenotype in Humans using Ensembl Resources (DECIPHER) (https://www.deciphergenomics.org/browser) and the Clinical Genome Resource (ClinGen) (http://www.ncbi.nlm.nih.gov/projects/dbvar/clingen/) were used to determine the pathogenicity of candidate CNVs. The clinical significance of the candidate CNVs was evaluated according to the criteria of the ACMG and the ClinGen [23].

### Chromosomal microarray analysis

To validate the CNV found by trio WES, chromosomal microarray analysis (CMA) was performed for the proband using an Affymetrix Cytoscan 750K GeneChip. The procedure was performed according to the manufacturer’s instruction. Data analysis was performed using the Chromosome Analysis Suite (ChAS) 4.1 software. CNVs larger than 100 kb or those that affected more than 50 contiguous probes were considered. The pathogenicity evaluation of candidate CNVs referred to the method described in the trio WES section above.

### Testing for fragile X syndrome, validation and pedigree analysis

The CGG repeats in the 5′-untranslated region of the *FMR1* gene were detected by fluorescence polymerase chain reaction products [24]. Gap-PCR was used to verify the diagnostic deletion, following the manufacturer’s protocol using LA Taq DNA polymerase (TaKaRa, Chiba, Japan). Forward and reverse primers were designed flanking the deletion breakpoints (forward: 5’ GCTTGAGGACAGTAATCACATC 3’; reverse: 5’ GCCTGTAGTCTGATTGCCA 3’). The gap-PCR products were electrophoresed, then sequenced in Tianyi Huiyuan Biotechnology Co., Ltd. (Wuhan, China), and the deletion breakpoints were validated by direct Sanger sequencing.

## Results

The triplet repeat number of CGG in the *FMR1* gene of the proband’s elder son was 25, which is in the normal range (6 to 44 CGG; Supplemental Figure S1) [24]. It partially demonstrated that the NDD phenotype in this family is not related to fragile X syndrome. Subsequently, we evaluated the filtered genetic variants detected by trio WES. Several rare nonsynonymous variants in NDDs-associated genes were identified; these are detailed in Supplemental Table S1. Three rare, maternally-inherited missense variants were identified in genes associated with autosomal dominant conditions that were classified as either benign or likely benign. Other heterozygous variants were in genes associated with autosomal recessive diseases. Based on the pathogenic classification of variants and the inheritance model, none of the nonsynonymous variants were considered to contribute to the phenotype. Besides, the trio WES analysis revealed a maternally inherited 1.31 Mb deletion at 1p13.2 (GRCh37/hg19 chr1: 113,633,912–114,944,107) in the proband. No nonsynonymous hemizygous variants were identified in the deletion region and the putative deletion was confirmed by CMA (Fig. 1D).

As depicted in the electrophoresis diagram (Fig. 1B), the presence of an 836 bp band indicated that the corresponding NDD patients of the family carried this deletion. All five symptomatic patients, including the proband (II-3), her mother (I-2), her younger brother (II-5) and her two sons (III-2 and III-3), carried the same heterozygous deletion, while the deletion was absent in the other three healthy individuals (I-1, II-1 and II-4). As shown in Fig. 1C, the accurate breakpoints of the deletion were identified by Sanger sequencing, by which the size of the deletion was 1,408,072 bp (chr1:113,536,526–114,944,597).

No CNVs fully encompassing the diagnostic 1p13.2 deletion in our cases were recorded in the DGV up to November 11, 2022. However, within the 1,408,072 bp deletion of 1p13.2 identified, one potentially benign CNV larger than 1 Mb in size was found in two individuals in the DGV (accession number: esv23869). According to the data in the DECIPHER database, the deleted 1p13.2 region covered 12 protein-coding genes, which were all listed in Online Mendelian Inheritance in Man (OMIM) database (Supplemental Table S2). Among them, three genes were human genetic disease-associated: *AP4B1*, associated with autosomal recessive spastic paraplegia 47 (MIM # 614,066), *LRIG2*, associated with autosomal recessive urofacial syndrome 2 (MIM # 615,112), and *PTPN22*, associated with susceptibility to diabetes type 1 (MIM # 222,100), rheumatoid arthritis (MIM # 180,300), and systemic lupus erythematosus (MIM # 152,700). Besides, the diagnostic deletion region encompassed *HIPK1* and involved parts of *TRIM33*. The DECIPHER database indicates that these two genes are potentially haploinsufficient, yet there are no definitive human diseases associated with them in the OMIM database (retrieved February 2, 2023).

## Discussion and conclusions

NDDs are characterized by a broad range of conditions that impact brain development and produce impairments of functioning. Although several contributing factors, such as prenatal exposure to the drug, virus infection and toxic agents, can lead to congenital NDDs, genetic factors are widely considered to be the most common causes. Unfortunately, due to the complex genetic heterogeneity of NDDs, it is still sometimes difficult to identify the underlying genetic cause. WES outperformed traditional methods in the detection of the prevalent types of pathogenic variants (SNVs and Indels) for NDDs, and has been widely used for genetic testing for patients with NDDs [25, 26]. Nowadays, the combination of variant screening and CNV calling simultaneously was increasingly used in WES, which largely improved the detection efficiency for the diagnosis of NDDs. Here, we reported a proband with NDD, who carried a novel 1.41 Mb heterozygous deletion located at 1p13.2 identified by trio WES. Besides, no pathogenic or likely pathogenic SNVs/Indels associated with her observed phenotypes were detected by trio WES.

We sought to evaluate associations between the 1p13.2 deletion and NDDs. To the best of our knowledge, only five articles have reported 1p13.2 deletions in five patients [11–15]. Additionally, 14 individuals with chromosomal 1p13.2 deletions (0.17-18.88 Mb) partially overlapping the deletion region of our case have been documented in the DECIPHER database (retrieved November 11, 2022), and one of them (DECIPHER patient ID: 274,660) was reported by Linhares et al. in the previous literature [14]. The detailed phenotypes of the reported 18 individuals were summarized in Table 1. To perform a genotype-phenotype correlation study of 1p13.2 deletions, eight previously described patients were excluded for further analysis, including four patients (P5, P6, P7 and P9) carrying double genetic CNVs or pathogenic SNVs, and four patients (P12, P14, P15 and P18) without any NDDs-associated phenotypes recorded in the DECIPHER database. We compared the clinical characteristics of our patients to the remaining 10 patients (Table 2). As shown, a total of 9 out of 10 (90%) patients were described with intellectual disability, 8 (80%) with language impairments, and 8 (80%) with gross motor developmental delay. Besides, 50-60% of cases were observed with short stature, facial appearance and neck abnormality. Such features have occurred in Noonan Syndrome, which is also characterized by developmental delay, intellectual impairment, short stature, and distinctive facial features, such as low-set ears, hypertelorism and ptosis [14]. The deletion region of all the 10 preceding cases encompassed the *NRAS* gene. The previous studies suggested that such features similar to Noonan Syndrome may be potentially attributed to the haploinsufficiency of the *NRAS* gene [11, 14]. The definitive association between alteration of the *NRAS* gene and Noonan syndrome has been demonstrated by the ClinGen Intellectual Disability and Autism Gene Curation Expert Panel. However, the diagnostic 1.41 Mb deletion (chr1:113,536,526–114,944,597) at 1p13.2 in our cases contains 12 protein-coding genes, excluding *NRAS*. As the proband’s phenotype described above, she also had an intellectual disability, language impairments, a mild facial appearance and a webbed neck, but did not present short stature. It suggested that the phenotypes of our cases could not be explained by the haploinsufficiency of the *NRAS* gene. We focused on the overlapping deletion regions at 1p13.2 between the 10 patients included in the further analysis and our patient. As illustrated in Fig. 1E, except that the deletion region of P3 was relatively unclear, the overlapping region extends from 113,709,879 to 114,944,597 at 1p13.2, covering 11 genes (Supplemental Table S2). As far as we can see, based on inheritance patterns and clinical correlations, the haploinsufficiency of the genes *AP4B1*, *LRIG2*, *PTPN22* and *HIPK1* may not be thought to lead to the phenotype of this family. To the highest degree of our knowledge, only two studies have suggested that *TRIM33* might be a candidate gene associated with autism and that the decreased TRIM33 gene expression might be correlated with autism symptoms [27, 28]. Despite evidence suggesting a potential correlation between the haploinsufficiency of *TRIM33* and the development of autism, a kind of NDDs, the correlation is yet to be established. Nevertheless, the phenotypes of our patients could not be fully explained by autism. It stays inconclusive whether the overlapping region contributes to the patients’ phenotypic characteristics.

**Table 1:** Clinical features of 19 patients with chromosomal deletions partially overlapping 1p13.2

Patients (Gender)	Chr. Band (hg19: Chr. position)	Size (Mb)	Other genetic CNVs or SNVs	Short stature	Neck abnormality	Facial features	Intellectual disability	Language impairments	Delayed gross motor development	Epilepsy	Behavioral problems	Inheritance	*NRAS* deleted	Reference/ Patient ID
P1 (M)	1p13p22.3 (NR)	NR	No	-	Short neck	+	ND	+	+	ND	ND	de novo	+	15
P2 (F)	1p13.1p21.1 (Max.: chr1:102,446,359–116,491,030; Min.: chr1:103,804,508–115,747,737)	11.9–14.0	No	+	Webbed neck	+	+	+	+	+	ND	de novo	+	[12]
P3 (F)	1p13.1p13.2 (Min.: chr1:114,609,456–116,035,987)	1.4–3.1	No	ND	ND	+	+	+	+	ND	+	de novo	+	[13]
P4 (M)	1p13.2 (chr1:112,096,417–115,805,157)	3.71	No	+	Broad neck	+	+	+	+	ND	ND	de novo	+	[14] /Patient ID: 274,660
P5 (M)	1p13.2 (NR)	5.3	*SCN8A* c.3967G > A (het)	+	ND	+	+	+	+	+	ND	de novo	+	11
P6 (M)	1p13.2 (chr1:114,012,621–114,185,094)	0.172	2p12 (chr2:78,495,678–78,716,453) dup	ND	ND	ND	ND	ND	ND	ND	ND	unknown	-	Patient ID: 371,208
P7 (M)	1p13.2 (chr1:114,189,096–114,733,279)	0.544	16q22.2 (chr16:72,348,999–72,733,544) del	ND	ND	ND	ND	ND	ND	ND	+	unknown	-	Patient ID: 288,445
P8 (F)	1p13.2 (chr1:113,525,828–115,713,872)	2.19	No	-	ND	+	+	+	+	ND	ND	de novo	+	Patient ID: 258,063
P9 (NR)	1p13.2 (chr1:113,377,785–115,715,743)	2.34	19p12 (chr19:23,255,525–23,689,288) del	ND	ND	+	+	ND	ND	ND	ND	de novo	+	Patient ID: 289,046
P10 (M)	1p13.1p13.2 (chr1:113,268,038–117,119,460)	3.85	No	ND	Short neck	ND	+	+	+	ND	ND	de novo	+	Patient ID: 253,793
P11 (F)	1p12p13.2 (chr1:113,709,879–118,344,568)	4.63	No	+	ND	+	+	ND	ND	ND	ND	unknown	+	Patient ID: 351,409
P12 (M)	1p13.1p13.3 (chr1:111,146,019–116,382,675)	5.24	No	ND	ND	ND	ND	ND	ND	ND	ND	unknown	+	Patient ID: 305,699
P13 (F)	1p11.2p13.2(chr1:112,633,589–121,281,170)	8.64	No	+	ND	ND	+	+	+	ND	ND	unknown	+	Patient ID: 295,430
P14 (M)	1p13.2p21.1 (chr1:102,734,224–114,766,687)	12.03	No	ND	ND	ND	ND	ND	ND	ND	ND	unknown	-	Patient ID: 472,268
P15 (M)	1p13.1p21.1 (chr1:103,782,322–117,597,970)	13.82	No	ND	ND	ND	ND	ND	ND	ND	ND	de novo	+	Patient ID: 281,269
P16 (F)	1p12p21.1 (chr1:105,976,094–120,529,725)	14.55	No	+	Short neck	ND	+	ND	+	ND	ND	de novo	+	Patient ID: 250,335
P17 (F)	1p12p21.2 (chr1:102,021,465–119,737,478)	17.71	No	ND	ND	ND	+	ND	ND	+	ND	de novo	+	Patient ID: 428,943
P18 (F)	1p13.2p21.3 (chr1:94,791,819–113,667,311)	18.88	No	ND	ND	ND	ND	ND	ND	ND	ND	de novo	-	Patient ID: 279,246
P19 (F)	1p13.2(chr1:113,536,526–114,944,597)	1.41	No	-	+	+	+	+	ND	-	-	maternally inherited	-	Current study

Chr., Chromosome; M, male; F, female; +, present; -, absence; NR, not reported; ND, not determined; Max., the maximum size of the deletion; Min., the minimum size of the deletion; het, heterozygous; dup, duplication; del, deletion

**Table 2:** Summary of the clinical features of our patients and the 10 previously reported patients

	Current study					Previous studies
	The proband (II-3)	Her mother (I-2)	Her younger brother (II-5)	Her elder son (III-2)	Her younger son (III-3)	
**Gender**						
Female	Yes	Yes	No	No	No	7 (70%)
***NRAS*** **gene deleted**	No	No	No	No	No	10 (100%)
**Short stature**	No	No	No	No	No	5 (50%)
**Facial appearance**						6 (60%)
Full eyebrows	Yes	NP	NP	Yes	Yes	1 (10%)
Hypertelorism	Yes	NP	NP	Yes	Yes	2 (20%)
Ptosis	Yes	NP	NP	Yes	Yes	2 (20%)
Low-set ears	Yes	NP	NP	Yes	Yes	3 (30%)
Upturned nose	Yes	NP	NP	Yes	Yes	1 (10%)
Overbite	Yes	NP	NP	Yes	Yes	0
Open mouth	Yes	NP	NP	Yes	Yes	1 (10%)
Neck abnormality	Yes	NP	NP	Yes	Yes	5 (50%)
**Neurodevelopmental disorder**						
Intellectual disability	Yes	Yes	Yes	Yes	Yes	9 (90%)
Language impairments	Yes	Yes	Yes	Yes	Yes	8 (80%)
Gross motor developmental delay	Unknown*	Unknown*	Unknown*	Unknown*	Unknown*	8 (80%)
Epilepsy	No	No	No	No	No	2 (20%)
Behavioral problems	No	No	No	No	No	1 (10%)

NP, The clinical phenotypes was not provided by the proband’s family; *, The patients in the current study could sit, walk and run without support, but their gross motor milestones were unclear

Many studies have demonstrated that recurrent CNVs are common causes of NDDs and are associated with a constellation of neurodevelopmental traits [5, 29]. Nevertheless, non-recurrent CNVs are also significant pathogenic factors of NDDs. Nearly 5.1% of patients with ID, DD, ASD and multiple congenital abnormalities were found to have a single non-recurrent rare CNV, as well as 7.1% of patients had a known recurrent CNV [30]. Among the previously reported 10 individuals, de novo deletions have been observed in 8 patients (80%), and the inheritances of the remaining two are yet unknown. The 1p13.2 deletion found in our patient was inherited from her mother who had similar phenotypes. Furthermore, no recurrent deletions were reported in the region of 1p13.2, indicating that this region is not prone to hotspot deletion.

In the present study, we first identified a novel 1.41 Mb heterozygous 1p13.2 deletion with precise breakpoints in five patients in a three-generation Chinese family. The 1p13.2 deletion segregates in the NDDs family. Therefore, that may provide strong evidence to support the pathogenicity of the 1p13.2 deletion. It suggested that the NDD phenotype of our patients could be explained by the 1.41 Mb 1p13.2 deletion. Our study might supplement the spectrum of 1p13.2 deletion-NDDs. However, further in-depth functional experiments are still needed to establish a genotype and phenotype relationship.

## Electronic supplementary material

Below is the link to the electronic supplementary material.

Supplementary Material 1

## Data Availability

The raw sequence data reported in this paper have been deposited in the Genome Sequence Archive (Genomics, Proteomics & Bioinformatics 2021) in National Genomics Data Center (Nucleic Acids Res 2022), China National Center for Bioinformation / Beijing Institute of Genomics, Chinese Academy of Sciences (GSA-Human: HRA003956) that are publicly accessible at https://ngdc.cncb.ac.cn/gsa-human.
