# Supplementary material for: A novel 1p13.2 deletion associates with neurodevelopmental disorders in a three-generation pedigree
Source: BMC Med Genomics. 2023 May 23;16:114. doi: 10.1186/s12920-023-01534-7 (PMC10207759; doi:10.1186/s12920-023-01534-7)
Supplement: Supplementary file 1 — Supplementary Material 1 [file 12920_2023_1534_MOESM1_ESM.docx]

**Supplementary Materials**

**Supplemental method**

**Variants filtering steps.**

The annotated variants were filtered through a stepwise in-house protocol as follows.

First, the known pathogenic variants step. The known or reported pathogenic variants were identified by comparing the variants recorded in the Human Gene Mutation Database (HGMD) (www.hgmd.cf.ac.uk) and ClinVar (https://www.ncbi.nlm.nih.gov/clinvar).

Second, the genotype-driven step. The variants were prioritized based on sequencing quality, minor allele frequency (MAF) (autosomal dominant inheritance: MAF≤0.3%, autosomal recessive inheritance: MAF≤5%), variant types and in silico predictions. The minor allele frequency was obtained from the publicly available databases, such as gnomAD, 1000 genomes project, NHLBI Exome Sequencing Project 6500 (ESP6500), and Exome Aggregation Consortium (ExAC).

Third, the phenotype-driven analysis step. With the aid of HPO matching, the correlation between genotype and phenotype was evaluated. Candidate rare variants in the gene associated with human genetic diseases and potentially related to the patient’s clinical features were then screened.

Finally, the manual analysis step. The variants would be further investigated by trained genetic professionals based on inheritance patterns, clinical correlations (mainly referred to OMIM database and GeneReviews) and relevant literature (mainly referred to PubMed and mastermind).

**Supplemental Table S1.** **Overview of the rare nonsynonymous variants detected by trio WES.**

| **Gene** | **Zygosity** | **Chromosome Position (GRCh37/hg19)** | **cDNA Change (Amino Acid Change)** | **OMIM Disease** | **Inheritance** | **Allele**  **Frequency*** | **Damaging Score**** | **ACMG Classification** | **Reasons for exclusion** |
| --- | --- | --- | --- | --- | --- | --- | --- | --- | --- |
| *KAT6A* | Heterozygous | chr8:41791254 | c.4484G>A (p.Arg1495His) | Arboleda-Tham syndrome (AD) | Maternal | 0.0027 | 0.65 | B | classified as benign: allele frequency = 0.0027 is greater than the threshold 0.0001 (BS1); allele count = 51 in the gnomAD exomes (BS2). |
| *CNOT3* | Heterozygous | chr19:54652445 | c.1373C>T (p.Pro458Leu) | Intellectual developmental disorder with speech delay, autism, and dysmorphic facies (AD) | Maternal | 0.0003 | 0.22 | B | classified as benign: allele frequency = 0.0003 is greater than the threshold 0.0001 (BS1); allele count = 6 in the gnomAD exomes (BS2); benign computational verdict based on 18 benign predictions vs. 5 pathogenic predictions (BP4) |
| *KMT2C* | Heterozygous | chr7:151945297 | c.2222T>C (p.Met741Thr) | Kleefstra syndrome 2(AD) | Maternal | 0.0024 | 0.09 | LB | classified as likely benign: allele count = 47 in the gnomAD exomes (BS2); benign computational verdict based on 21 benign predictions vs. 2 pathogenic predictions (BP4) |
| *DYM* | Heterozygous | chr18:46783425 | c.1415C>T (p.Ala472Val) | Dyggve-Melchior-Clausen disease (AR)/ Smith-McCort dysplasia (AR) | Paternal | 0.0011 | 0.96 | VUS | Inconsistent with the inheritance model |
| *OSGEP* | Heterozygous | chr14:20920589 | c.133dup (p.Thr45AsnfsTer58) | Galloway-Mowat syndrome 3 (AR) | Maternal | <0.0001 | NA | LP | Inconsistent with the inheritance model |
| *PRSS12* | Heterozygous | chr4:119204126 | c.2180T>C (p.Ile727Thr) | Intellectual developmental disorder, autosomal recessive 1 (AR) | Maternal | 0.0002 | 0.91 | VUS | Inconsistent with the inheritance model |
| *PUS7* | Heterozygous | chr7:105098381 | c.1850-8C>T | Intellectual developmental disorder with abnormal behavior, microcephaly, and short stature (AR) | Maternal | <0.0001 | NA | VUS | Inconsistent with the inheritance model |
| *VPS13B* | Heterozygous | chr8:100880603 | c.11302G>C (p.Gly3768Arg) | Cohen syndrome (AR) | Maternal | 0.0003 | 0.91 | VUS | Inconsistent with the inheritance model |
| *KIFBP* | Heterozygous | chr10:70775384 | c.1078A>G (p.Lys360Glu) | Goldberg-Shprintzen megacolon syndrome (AR) | Paternal | 0.0005 | 0.57 | VUS | Inconsistent with the inheritance model |

* Genome Aggregation Database (gnomAD) exomes East Asian allele frequency. ** damaging score was evaluated by VarCards. AD, autosomal dominant inheritance; AR, autosomal recessive inheritance; VUS, variant of uncertain significance; B, variant of benign; LB, variant of likely benign; LP, variant of likely pathogenic; NA, not available.

**Supplemental Table S2.** **Genes encompassed by the 1p13.2 deletion (chr1:113536526-114944597).**

| Gene | OMIM Disease |
| --- | --- |
| *AP4B1* | Spastic paraplegia 47 (AR) |
| *BCL2L15* | NA |
| *DCLRE1B* | NA |
| *HIPK1* | NA |
| *LRIG2** | Urofacial syndrome 2 (AR) |
| *MAGI3* | NA |
| *OLFML3* | NA |
| *PHTF1* | NA |
| *PTPN22* | {Diabetes, type 1, susceptibility to} (AR); {Rheumatoid arthritis, susceptibility to}; {Systemic lupus erythematosus susceptibility to} (AD) |
| *RSBN1* | NA |
| *SYT6* | NA |
| *TRIM33* | NA |

*The overlapping region do not cover *LRIG2* gene. AD, autosomal dominant inheritance; AR, autosomal recessive inheritance; NA, not available.


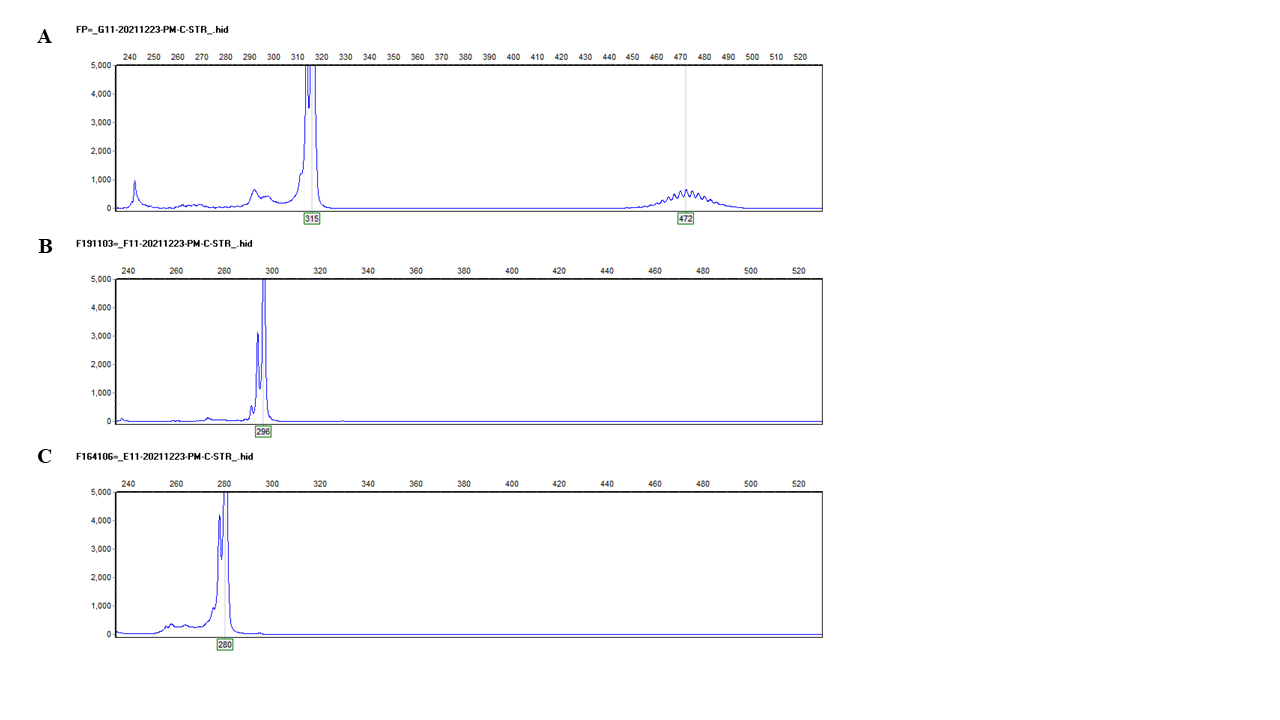


**Supplemental Figure S1** The results of the fluorescence analysis of polymerase chain reaction products for fragile X syndrome. (A) The female positive control with 38/99 CGG repeats in the *FMR1* gene. (B) The negative control with 31 CGG repeats. (C) The proband’s elder son with 25 CGG repeats.
